# Domiciliary High-Flow Nasal Therapy in Primary Ciliary Dyskinesia

**DOI:** 10.7759/cureus.34177

**Published:** 2023-01-25

**Authors:** Rita Gomes, Joana Queirós, Joana Borges, Ana Lúcia Cardoso, Telma Barbosa

**Affiliations:** 1 Pediatrics, Centro Materno Infantil do Norte Albino Aroso, Centro Hospitalar Universitário do Porto, Porto, PRT; 2 Pediatrics, Centro Hospitalar Vila Nova de Gaia/Espinho, Porto, PRT; 3 Pediatric Intensive Care Unit, Centro Materno Infantil do Norte Albino Aroso, Centro Hospitalar Universitário do Porto, Porto, PRT; 4 Pediatric Pneumology, Centro Materno Infantil do Norte Albino Aroso, Centro Hospitalar Universitário do Porto, Porto, PRT

**Keywords:** noninvasive mechanical ventilation, palliative care, high-flow nasal oxygen, respiratory insufficiency, primary ciliary dyskinesia (pcd)

## Abstract

We report the case of an adolescent with severe primary ciliary dyskinesia (PCD) phenotype associated with a rare genotype. His clinical condition deteriorated, with daily cough and breathlessness, hypoxemia, and lung function decline. Despite being started on home noninvasive ventilation (NIV), the symptoms progressed to dyspnea at rest and thoracic pain. High-flow nasal cannula (HFNC) was started during the daytime as an adjuvant to NIV, and he was started on regular oral opioids for pain and dyspnea control. There was a clear improvement in comfort and dyspnea and breathing work relief. Additionally, better exercise tolerance was also noted. He is currently on the lung transplant waiting list. We aim to emphasize the benefits of HFNC as an add-on therapy for the management of chronic breathlessness since our patient experienced an improvement in breathing and exercise tolerance. However, there is a paucity of studies regarding domiciliary HFNC, particularly in pediatric age. Therefore, further studies are needed to achieve optimal and personalized care. Close monitoring and frequent reassessment in a specialized center are key to adequate management.

## Introduction

Primary ciliary dyskinesia (PCD) is a rare syndrome with a reported prevalence of one in 10,000-20,000 liveborns, charac­terized by genetic heterogeneity and clinical variability [1-4]. As a result of mucociliary clearance disturbance by an impaired motile cilia function, children with severe PCD usually present early in life with neonatal respiratory distress, persistent sinonasal and middle ear disease, chronic daily cough, and bronchiectasis [2].

However, disease progression is highly variable, with some patients maintaining reasonably good pulmonary function and quality of life, whereas other patients have worse outcomes. A few studies have suggested an association between some genes and the severity of pulmonary disease, but the evidence remains scarce, and prospective registries are needed to properly understand these associations [1].

Successful management of children with PCD requires a multidisciplinary specialized team [5]. Due to a lack of international consensus, the current approach is largely based on cystic fibrosis (CF) guidelines and focuses on careful monitoring, physiotherapy, and treatment of infections to delay pulmonary function decline while maintaining patients’ health and psychological well-being [1,5].

## Case presentation

We present the case of a 14-year-old male with a severe phenotype of PCD. He was born to consanguineous parents, and there was no other relevant family background. He presented with unexplained episodes of desaturation during the neonatal period. Since he was seven-month-old, he had daily rhinorrhea, productive cough with mucopurulent sputum, and digital clubbing, and at the age of three, failure to thrive and bronchiectasis. Until the age of six, he had multiple respiratory infections, some requiring hospital admission and intravenous antibiotic treatment, and underwent myringotomy and adenoidectomy for bilateral chronic serous otitis media complicated with hearing loss. He was then referred to our center for further investigation.

**Figure 1:**
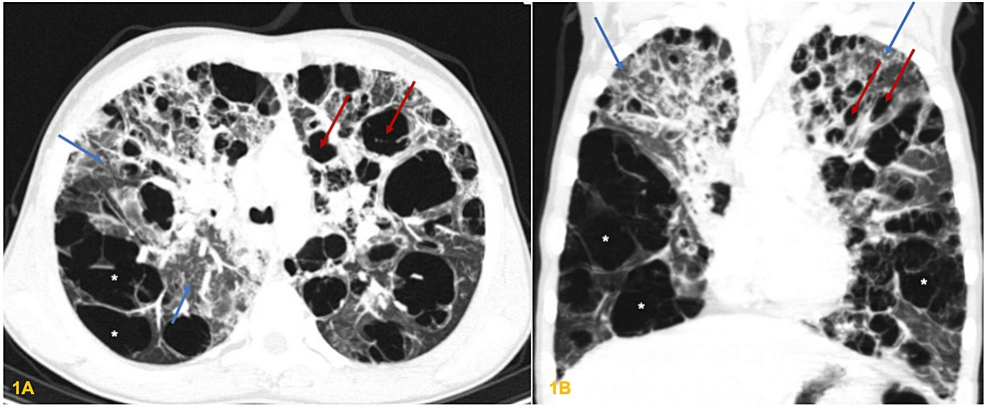
CT scans showed centrilobular and paraseptal emphysema (stars), extensive varicoid and cystic bronchiectasis (red arrows), mucoid impaction, and ground glass opacification areas (blue arrows). CT: computed tomography

Computed tomography (CT) scans showed centrilobular and paraseptal emphysema, extensive varicoid and cystic bronchiectasis, mucoid impaction, and ground glass opacification areas (Figure 1A and Figure 1B). Bronchial fibroscopy revealed inflammation of bronchial airways with normal bronchoalveolar lavage. Pulmonary function tests (PFTs) revealed a forced expiratory volume in the first second (FEV1) of 55.5% and forced vital capacity (FVC) of 71.9%. Next-generation sequencing (NGS) of cystic fibrosis transmembrane conductance regulator (*CFTR*) gene and genes associated with pulmonary surfactant metabolism dysfunction (*ABCA3*, *SFTPB*, and *SFTPC*) identified no pathogenic mutations. Sweat tests, serum levels of alpha-1-antitrypsin, and immunological studies were also normal, including immunoglobulins, oxidative burst test, serum, and bronchoalveolar lymphocyte subset immunophenotyping and serum autoantibodies (namely, antinuclear, anti-saccharomyces cerevisiae, antineutrophil cytoplasmic antibodies, rheumatoid factor, anti-proteinase 3, and anti-myelin oligodendrocyte glycoprotein). Electronic microscopy of bronchial mucosa revealed the absence of the inner arm of the dynein in more than 80% of the peripheral microtubules, which was compatible with PCD. Subsequently, massive parallel sequencing of 27 genes associated with PCD was performed and identified a homozygous c.455G>A p.(Arg152His) variant in the *CCDC114* gene and a heterozygous c.2297A>G p.(Glu766Gly) variant in the *SPAG1* gene. Molecular studies of the parents showed that they were both carriers of the *CCDC114* gene variant.

A multidisciplinary follow-up, including palliative care support, was maintained during the following years. He was started on regular respiratory physiotherapy and was treated with caloric and vitamin supplements, acetylcysteine, bronchodilators, azithromycin (three times a week), inhaled corticosteroids, and supplemental oxygen. By the age of 12, his clinical condition deteriorated, presenting daily symptoms (cough and breathlessness on exertion). The 24-hour oximetry and arterial blood gases had evidence of persistent hypoxemia, despite oxygen supplementation, and the PFT showed declining pulmonary function (FEV_1_: 35.4%, FVC: 60.6%). Electrocardiogram and cardiac ultrasound revealed no indirect signs of pulmonary hypertension or cor pulmonale. He was then started on home noninvasive ventilation (NIV) during sleep periods and was referred for evaluation by the lung transplantation team.

Over the past two years, his symptoms worsened, with dyspnea at rest and occasional thoracic pain. Capnography showed a partial pressure of carbon dioxide (PaCO_2_) of 47 mmHg. High-flow nasal cannula (HFNC) was started during the daytime (flow: 30 L/minute, fraction of inspired oxygen (FiO_2_): 0.32), maintaining NIV during sleep and conventional oxygen therapy at school, and he was started on regular oral opioids for pain and dyspnea control. There was a clear improvement in comfort and reduction of dyspnea and breathing work. Additionally, better exercise tolerance was also noted: he was able to slowly walk small distances, although having difficulties climbing stairs or walking fast. The 24-hour oximetry also revealed an increase in mean oxygen saturation (SpO_2_) (up to 97%) and a reduction in mean heart rate (93 bpm). Considering the PFT showed an FEV_1_ of less than 40% and there were markers of shortened survival (hypoxemia and hypercarbia), he was proposed for a lung transplant. He is currently 14 years old and is on the lung transplant waiting list.

## Discussion

We report the case of an adolescent with a severe PCD phenotype associated with a rare genotype. More than 50 disease-causing genes have been identiﬁed so far, the most frequently reported being *DNAH5 *and *DNAH11 *[6]. Nevertheless, in up to 30% of individuals with PCD diagnosis, there is no identifiable genetic cause. Recently, genotype-phenotype studies have demonstrated that patients with certain variant alleles of *CCDC39*, *CCDC40*, *MCIDAS*, and *CCNO *exhibit a much more aggressive disease course, with poor pulmonary function and rapid progression to lung failure, whereas patients with certain variants of *DNAH9* often have mild respiratory dysfunction or even normal lung function [3,6]. Despite being rare, *CCDC114* gene variants have been described in patients with PCD, who present high clinical heterogeneity [5,7,8]. This case extends the phenotype spectrum of the *CCDC114* gene. However, the contribution of the genetic variant p.(Arg152His) to this severe and atypical phenotype should be reviewed in the future.

The benefit of HFNC in patients with PCD has not been established. This treatment is characterized by a gas delivery system that provides heated, humidified air or supplemental oxygen on a high-flow nasal cannula, improving gas exchange and reducing the work of breathing [9,10]. The mechanism of action is multifactorial: heating and humidification improve ciliary function and mucus hydration, ensuring optimal mucociliary clearance; the positive airway pressure exerted by the high flow improves alveolar recruitment, increases tidal volume, reduces breathing work, and improves dead-space washout [10,11]. Recently, it has evolved from a treatment for hospitalized patients to a possible add-on therapy for domiciliary use in patients with chronic respiratory diseases [12].

The role of HFNC in airway disease has primarily focused on chronic obstructive pulmonary disease (COPD), albeit being increasingly used for both children and adults with acute hypoxemic respiratory failure [9]. Previous studies reported a decrease in the frequency of exacerbations and improvement in FEV_1_ in patients with obstructive lung diseases and mucus retention [13]. Other studies in patients with advanced COPD and chronic hypoxemic respiratory insufficiency have demonstrated an improvement of respiratory effort with a reduction in respiratory rate and PaCO_2_, as well as an increased exercise performance [14-16]. More recently, some authors described the advantages of HFNC as an adjunct to long-term oxygen therapy, significantly reducing exacerbations and hospitalizations, particularly in those with frequent exacerbations [12,17]. Moreover, humidification therapy offers a promising management approach for patients with bronchiectasis, improving mucociliary clearance and therefore reducing airway inflammation and recurrent infections [12]. HFNC was also found to significantly improve quality of life compared with conventional care, even when used for short periods of time [12,14].

Given its beneﬁts in the acute setting, the convenience of use, and comfort for patients, domiciliary HFNC is a growing area of interest [9]. Although there is limited experience regarding HFNC use in end-of-life patients and palliative care, emerging evidence suggests that it may have a role in alleviating chronic breathlessness, respiratory distress, and failure despite optimal treatment [7-17]. In some patients requiring oxygen supplementation, especially those with excessive secretions, HFNC may be a preferred alternative oxygen-delivering method, since conventional oxygen therapy improves oxygenation but may not effectively decrease dyspnea [18].

## Conclusions

With the presentation of this case, we intend to describe the benefits of HFNC as an add-on therapy for the management of severe chronic breathlessness in a pediatric patient with severe PCD, who experienced improvement of breathing and exercise tolerance, with a positive impact on quality of life. The use of HFNC, as described in this case, is supported by recent studies conducted on chronic respiratory diseases. However, there is still limited experience in the domiciliary use of HFNC, particularly in the pediatric age. Although there are reports of improvement in mucociliary clearance and ventilation in patients with chronic mucus hypersecretion and bronchiectasis, there is a scarcity of literature investigating the use of HFNC in patients with PCD. Therefore, further studies are needed to assess which patients will particularly benefit from HFNC to achieve optimal and personalized medicine. Close monitoring and frequent reassessment in specialized PCD centers are key to adequate management.
